# Motor action observation evaluation questionnaire: development and initial validity evidence for the scores in people with and without chronic low back pain

**DOI:** 10.3389/fpsyg.2026.1887879

**Published:** 2026-10-15

**Authors:** Roy La Touche, Prado Silván-Ferrero, Encarnación Nouvilas-Pallejá, Alba Paris-Alemany, Alfonso J. López-Rivero, Miguel A. Sorrel

**Affiliations:** 1 Programa de Doctorado en Psicología, Escuela de Doctorado, Universidad Autónoma de Madrid, Madrid, Spain; 2 Pain, Mental Health, Exercise and Technology Research Group (PAIN+MET), Faculty of Physiotherapy and Nursing, Universidad de Castilla-La Mancha, Toledo, Spain; 3 Department of Social and Organizational Psychology, National University of Distance Education, Madrid, Spain; 4 Department of Basic Health Sciences, Universidad Rey Juan Carlos, Alcorcón, Spain; 5 Physical Activity and Health Research Group (AFYS), Faculty of Health Sciences, Rey Juan Carlos University, Madrid, Spain; 6 Computer Science Faculty, Universidad Pontificia de Salamanca, Salamanca, Spain; 7 Departamento de Psicología Social y Metodología, Universidad Autónoma de Madrid, Madrid, Spain

**Keywords:** action observation, chronic low back pain, motor imagery, psychometrics, questionnaire development, rehabilitation

## Abstract

**Introduction:**

Chronic low back pain (CLBP) is highly prevalent and associated with functional limitations. Although action observation is increasingly used as a cognitive–motor rehabilitation strategy, no brief instrument exists to assess how individuals appraise and understand movements during observation.

**Methods:**

The Questionnaire for the Assessment of Motor Action Observation (CENOAM) was developed using a sequential mixed-methods design. Phase 1 involved item generation based on literature review and qualitative analysis, followed by expert content validation using Aiken’s *V*. Phase 2 consisted of a cross-sectional study including 280 participants (140 with CLBP and 140 asymptomatic). Dimensionality and psychometric properties of the CENOAM scores were examined using exploratory factor analysis, complementary dimensionality methods, internal consistency (*α*, *ω*), floor and ceiling effects, concurrent associations, and known-groups comparisons, following COSMIN recommendations.

**Results:**

The final seven-item instrument showed a parsimonious two-dimensional structure, comprising Familiarity and Competence–Demand, supported by converging evidence across multiple methods. Internal consistency was high for both total and subscale scores, and no floor or ceiling effects were observed. CENOAM scores were positively associated with motor imagery ability, physical activity, and pain self-efficacy, and negatively associated with fear of movement. Participants with CLBP showed significantly lower scores than asymptomatic individuals across all dimensions, with large effect sizes.

**Conclusion:**

The CENOAM provides a brief and interpretable measure of movement appraisal during action observation. Its scores show initial evidence of validity and may support the individualization of action-observation–based rehabilitation in musculoskeletal conditions.

## 1 Introduction

Chronic pain is a major public health problem with substantial individual and societal consequences (Fayaz et al., 2016). Within this broader context, chronic low back pain (CLBP) is highly prevalent and strongly associated with disability and reduced functioning (Ferreira et al., 2023; Vos et al., 2012). Because most cases are classified as non-specific (Bardin et al., 2017), contemporary management increasingly emphasizes not only biological mechanisms but also psychosocial and cognitive factors that may contribute to persistence, disability, and maladaptive behavioral responses (Côté et al., 2008; Salvetti et al., 2012; Wynne-Jones et al., 2008).

Among cognitive-motor approaches of growing interest in rehabilitation, action observation has received increasing attention. Action-observation training involves the systematic viewing of another person’s movements, usually presented from egocentric or allocentric perspectives, while engaging processes such as attentional selection, analysis, interpretation, and understanding of the observed action (La Touche, 2020). From a neurophysiological perspective, action observation is thought to elicit internal motor simulation of the visual sequence, producing patterns of brain activation that partially overlap with those involved in motor planning and execution (Buccino, 2014). This internal simulation, commonly described in terms of motor resonance, constitutes one of the mechanisms through which the action-observation network actively processes observed actions.

Importantly, the active nature of action observation does not imply an invariant or purely stimulus-driven response. Motor resonance and the broader processing of observed actions are modulated by both individual and contextual factors, including previous motor experience, movement familiarity, contextual congruence, expectations, and the meaning attributed to the observed action. Familiarity and contextual congruence facilitate motor resonance, whereas incongruence may attenuate it (Amoruso et al., 2016; Bianco et al., 2024). Likewise, the action-observation network shows greater engagement during observation of familiar actions, particularly in inferior parietal and temporal regions (Gardner et al., 2015), and affective or moral valence may also modulate neural responses (Craighero and Mele, 2018). Together, these findings support the view that action observation involves active, context-sensitive sensorimotor and interpretative processing shaped by prior experience, expectations, and goals (Chye et al., 2024; Suso-Martí et al., 2019, Welsh et al., 2009).

This issue may be particularly relevant in CLBP. Previous studies using images or videos have shown that fear of pain, pain-related fear, and kinesiophobia are associated with avoidance and altered appraisal of everyday actions (Demoulin et al., 2013; Houben et al., 2005; Pérez-Fernández et al., 2015; Trost et al., 2009). In addition, CLBP has been associated with enhanced activation of fear-related neural networks during motor anticipation (Meier et al., 2016) and with broader alterations in the processing of action-related information (Cracco et al., 2019; Hotta et al., 2017). Together, these findings suggest that the appraisal and interpretation of observed movements may be influenced by pain-related cognitive and affective processes in individuals with CLBP, supporting the need for methods capable of characterizing these subjective responses during action observation.

However, to our knowledge, there is currently no brief and clinically applicable instrument specifically designed to assess the subjective appraisal of observed movements across dimensions such as familiarity, perceived competence, movement interpretation, and perceived demand in a form applicable to both research and rehabilitation settings.

In response to this gap, we developed the Questionnaire for the Assessment of Motor Action Observation (Cuestionario de Evaluación de la Observación de Acciones Motoras; CENOAM) and sought initial validity evidence for its scores. The aims of this study were: (1) to generate questionnaire items representing the cognitive-affective components considered most relevant during action observation in rehabilitation contexts; and (2) to evaluate the psychometric properties of the resulting scores, including their internal structure, relations with external constructs, and known-groups validity in adults with CLBP and asymptomatic controls. A measure of this type may contribute to more individualized action-observation–based interventions by identifying appraisal-related barriers and potentially modifiable targets.

## 2 Methods

### 2.1 Study design

This study followed a sequential exploratory mixed-methods design, in which qualitative and quantitative data were independently collected and analyzed, followed by integration for interpretation (Guetterman et al., 2015). The research was conducted in two distinct stages: Phase 1 (Qualitative) and Phase 2 (Quantitative). The qualitative phase has been published separately (La Touche et al., 2026).

This study was approved by the bioethics committee of CSEU La Salle (CSEULS-PI-005/2020), and was conducted in accordance with the ethical principles outlined in the Declaration of Helsinki for research involving human subjects (World Medical Association, 2013).

In the second phase, a cross-sectional design was used to assess the psychometric properties of the CENOAM scores developed from Phase 1. Furthermore, known-groups validity was examined by comparing asymptomatic individuals and participants with chronic low back pain (CLBP). All score-validation procedures adhered to the COSMIN checklist for assessing the methodological quality of studies on patient-reported outcome measures (Mokkink et al., 2010a, b).

### 2.2 Participants

Patients with CLBP were recruited from two rehabilitation clinics. The inclusion criteria were: (a) low back pain for at least 6 months; (b) nonspecific low back pain; (c) pain intensity of 3 or higher on the numeric pain rating scale; (d) age between 18 and 65 years. Exclusion criteria included: (a) spinal surgery in the past year; (b) specific spinal pathologies such as malignancies or inflammatory diseases of joints and bones; (c) spinal trauma within the past year; (d) infectious or tumoral diseases; (e) uncorrected visual or cognitive impairments that could hinder audiovisual comprehension; (f) difficulties in comprehension or communication; and (g) insufficient knowledge of Spanish to follow the instructions for the variables collected.

Asymptomatic participants were recruited from the CSEULS community via social media posts, faculty bulletins, direct contact, and flyers. Eligible individuals included students, administrative or teaching staff, and members of their social networks. Inclusion criteria for this group were: (a) no pathologies or pain in the past 3 months; (b) age between 18 and 65 years. Exclusion criteria for this group were: (a) uncorrected visual or cognitive impairments precluding adequate comprehension of audiovisual material; (b) difficulties in comprehension or communication; and (c) insufficient Spanish proficiency to follow the instructions for the variables collected. All participants provided written informed consent before their enrollment in the study.

### 2.3 Procedures

The development of the questionnaire and the psychometric evaluation of the CENOAM scores followed a standardized seven-phase methodology (Artino et al., 2014): (1) an exhaustive literature review, (2) semi-structured interviews, (3) synthesis of the literature review and analysis of the semi-structured interviews (these three procedures were completed in Phase 1), (4) creation of questionnaire items, including item specification and identification of domains, (5) expert validation (content validity), (6) evaluation of the instrument’s comprehension and feasibility (cognitive interviewing) in a small patient group (pilot testing), and (7) psychometric evaluation of the CENOAM scores. All procedures were conducted between June 2022 and November 2024.

#### 2.3.1 Item development

Based on the integration of findings from the literature review and qualitative data obtained through semi-structured interviews followed by an interpretative phenomenological analysis, three researchers collaboratively defined the initial conceptual construct as the evaluation of perception, understanding, and self-efficacy during motor action observation. The literature informing this conceptualization included evidence concerning internal motor representation and action understanding during action observation (Buccino, 2014), the influence of previous motor experience and movement familiarity on the processing of observed actions (Calvo-Merino et al., 2006; Gardner et al., 2015), and the role of self-efficacy and related cognitive processes in observational learning (Bandura, 1977; Bandura and Jeffrey, 1973). These theoretical sources were integrated with the qualitative findings from Phase 1 to guide the initial specification of the construct and the subsequent development of questionnaire items. Subsequently, questionnaire items were drafted, and a preliminary pool of 11 items was developed through a structured consensus process (Jones and Hunter, 1995). After iterative refinement, 10 items were retained for the initial version of the questionnaire, categorized into four theoretical dimensions: (1) Perceived Demand, (2) Familiarity with Observed Motor Actions, (3) Competence, and (4) Fear and Avoidance of Motor Actions.

#### 2.3.2 Expert content validation

The preliminary 10-item list was submitted for content validation by an external panel of experts, who assessed each item in terms of relevance, exhaustiveness, and comprehensibility (content validation by judges). To be considered an expert judge, individuals had to meet the following criteria: (1) at least three publications on movement representation methods, (2) a minimum of 5 years of research experience in motor cognition or human movement sciences, (3) training and clinical experience in managing patients with chronic low back pain, and (4) knowledge of psychometric validation of questionnaires.

The expert content validation panel consisted of eight professionals (one physician, four physical therapists, and three psychologists), who assessed each item in terms of relevance, comprehensiveness, and comprehensibility. For each criterion, experts selected one of five verbal response categories: “strongly disagree,” “disagree,” “neither agree nor disagree,” “agree,” or “strongly agree.” Numerical values were not displayed to the expert judges. For quantitative analysis, responses were coded so that increasing values represented increasing agreement with item adequacy; consequently, higher Aiken’s *V* coefficients indicated greater expert agreement regarding content validity (Aiken, 1985).

Aiken’s *V* coefficients and their 95% confidence intervals were calculated separately for relevance, comprehensiveness, and comprehensibility. Confidence intervals were calculated using the score confidence interval procedure described by Penfield and Giacobbi (2004) and were used to quantify the precision and uncertainty surrounding the point estimates. The prespecified Aiken’s *V* < 0.70 threshold was applied to the point estimate as a screening indicator of potentially insufficient content validity rather than as an automatic item-deletion rule. Confidence-interval lower bounds were not subjected to the 0.70 threshold and were not used as criteria for item retention or removal. Final item-retention decisions were based on the combination of Aiken’s *V* point estimates and qualitative expert feedback concerning ambiguity, conceptual specificity, relevance, redundancy, and applicability.

#### 2.3.3 Cognitive interview

A cognitive-interviewing and pilot-testing procedure was conducted in a sample of 30 participants (15 asymptomatic individuals and 15 participants with CLBP). The procedure combined retrospective interviewing and verbal probing with numerical ratings of relevant aspects of each item; think-aloud interviewing was not used. Participants first completed the CENOAM and subsequently took part in a retrospective interview focused on item comprehension, interpretation of questions, response selection, uncertainty between response options, unclear wording, and general comments about the instrument (Farnik and Pierzchała, 2012). Verbal probing was additionally used to explore how participants interpreted specific terms or items and the reasoning underlying their responses. All interviews were conducted by one member of the research team with previous experience in cognitive interviewing.

Qualitative responses were documented using structured interviewer notes and subsequently reviewed by the research team to identify recurrent problems related to item comprehension, interpretation, wording, response processes, and administration. No formal qualitative coding framework was applied because the purpose of this stage was to identify practical or interpretative difficulties requiring refinement before the main psychometric study. Formal qualitative saturation was not used as a predefined criterion; instead, the recurrence of similar problems across participants was examined.

During the pilot study, participants were exposed to three different motor stimuli (trunk flexion, glute bridge, and squat). Pilot testing identified that some participants experienced difficulty providing a single global appraisal across different observed movements, because their perceptions and attributions could differ according to the specific action being observed. Based on this observation, the administration procedure for the main psychometric study was refined so that CENOAM responses referred to a single standardized motor stimulus rather than to a combined appraisal of multiple movements.

#### 2.3.4 Psychometric property evaluation

Following the theoretical development of the questionnaire, the psychometric properties of the CENOAM scores were evaluated. This included assessment of internal structure validity, floor and ceiling effects, concurrent validity, and known-groups (discriminant) validity by comparing responses between participants with CLBP and asymptomatic controls.

In the main psychometric study, CENOAM responses were obtained in relation to a single standardized observed motor stimulus. The selected movement consisted of a squat combined with evident forward trunk flexion. From a standing position, hip and knee flexion were initiated simultaneously during the descending phase, accompanied by forward flexion of the trunk. At the lowest position, the hips and knees remained flexed and the trunk remained flexed forward. The movement was completed by returning to the initial standing position through extension of the knees, hips, and trunk. In contrast to a conventional squat involving anterior trunk inclination with a relatively neutral spinal position, the stimulus incorporated a more evident lumbar and/or thoracic flexion component during descent.

The movement was recorded from an allocentric perspective using a general camera shot in the sagittal plane, allowing visualization of the complete motor action and trunk movement. Two equivalent versions of the stimulus were recorded, one performed by a young adult woman and the other by a young adult man. Both actors performed the same standardized movement. Participants were allowed to select which actor version they preferred to observe. Movement speed was standardized during recording using a metronome. Each repetition lasted approximately 2 s, comprising approximately 1 s for the descending phase (simultaneous hip and knee flexion with forward trunk flexion) and 1 s for the ascending phase (return to standing through knee, hip, and trunk extension).

The video was presented individually on a computer screen in a room without other participants and without recognizable visual or auditory distractions. Participants completed the CENOAM immediately after observing the standardized movement, with all items referring specifically to the movement they had just observed. If requested, participants were allowed to replay the stimulus up to two additional times before completing the questionnaire.

The CENOAM is not intended to assess a stable person-level trait independently of the observed action. Rather, its scores represent an individual’s appraisal or attribution in relation to a specific observed motor stimulus. Accordingly, the observed movement constitutes the explicit referent of the CENOAM items, and scores should be interpreted as movement-specific appraisals unless generalizability across different motor stimuli is demonstrated.

### 2.4 Sampling strategy and sample size

The sample size for the psychometric evaluation was determined based on exploratory factor analysis. A minimum of 280 participants was deemed necessary, assuming moderate conditions with communalities between 0.40 and 0.70 (Lloret-Segura et al., 2014). This estimation is consistent with methodological recommendations indicating that, even under optimal conditions, such as high communalities and clearly defined factor structures, factor analysis studies require over 200 cases (Ferrando Piera and Anguiano Carrasco, 2010; Lloret-Segura et al., 2014). Sampling was non-probabilistic (consecutive for the CLBP group; convenience for the asymptomatic group).

### 2.5 Data analysis

All analyses were conducted using jamovi v2.0 (Exploratory Factor Analysis module) and R. In R, the readxl and dplyr packages were used for data import and preparation, psych and EGAnet for psychometric and network analyses, and ggplot2 for data visualization. Categorical variables are presented as frequencies and percentages, and continuous variables as mean, standard deviation (SD), 95% confidence interval (CI), range, skewness, and kurtosis. Distributional properties were examined using histograms and Q–Q plots. For computation of the CENOAM total score, the two Perceived Demand items (Items 1 and 7) require reverse scoring so that higher scores consistently reflect a more favorable action-observation appraisal profile. In the final analytical dataset used in the present study, these items had already been recoded accordingly and were therefore stored in their final scoring direction.

Before conducting the additional analyses, CENOAM scoring was reverified directly against the final analytical dataset. The sum of the seven item scores, using the finalized item coding, reproduced the recorded CENOAM Total score exactly. No additional reverse-scoring transformation was therefore applied during this verification.

Given the inclusion of participants with CLBP and asymptomatic participants, item-level and CENOAM score correlation matrices were additionally examined separately by group.

#### 2.5.1 Construct validity—exploratory factor analysis (EFA)

To examine the internal structure of the CENOAM scores, an exploratory factor analysis (EFA) was conducted following current best-practice recommendations within the confirmatory–exploratory continuum (Lloret-Segura et al., 2014; Nájera et al., 2023). Because items were scored on an 11-point numerical scale (0–10) and treated as continuous, analyses were based on the Pearson correlation matrix, which is considered appropriate under these conditions.

Factor extraction was performed using the minimum residual (MINRES) method, which focuses on the common variance shared among items and has shown robust performance under non-normal conditions (Ferrando et al., 2022; Lloret-Segura et al., 2014). An oblique rotation (direct oblimin) was applied, allowing factors to correlate, as theoretically expected in psychological constructs. Factor interpretation was based on both pattern and structure matrices (Henson and Roberts, 2006).

Sampling adequacy was evaluated using the Kaiser–Meyer–Olkin (KMO) index and Bartlett’s test of sphericity. Dimensionality was assessed using a multimethod approach combining Horn’s parallel analysis (Horn, 1965), scree plot inspection, Velicer’s minimum average partial (MAP) test (Velicer, 1976), Very Simple Structure (VSS), Bayesian Information Criterion (BIC), sample-size adjusted BIC (Schwarz, 1978; Sclove, 1987), and Exploratory Graph Analysis (EGA) (Golino and Epskamp, 2017). Final decisions regarding the number of factors were based on convergence across methods, model parsimony, simple structure, and interpretability (Goretzko et al., 2021; Graña et al., 2026). The eigenvalue >1 rule was not used as a decision criterion.

Item retention was guided by a combination of statistical and theoretical criteria. Items were considered adequate when they showed a primary loading ≥0.40, communality ≥0.30 (or ≥0.10 as a minimum acceptable threshold in early-stage analyses), and no problematic cross-loadings, defined as secondary loadings ≥0.30 or a difference <0.20 between primary and secondary loadings (Costello and Osborne, 2005; Ferrando et al., 2022; Goretzko et al., 2021). Item complexity was additionally evaluated using Hofmann’s complexity index, with values >2 indicating potential multidimensionality (Hofmann, 1978). The prespecified loading, communality, and cross-loading thresholds were used as screening criteria rather than as automatic item-deletion rules. Item-retention decisions followed a multimethod and multicriterion approach in which primary and secondary factor loadings, communality, loading differentiation, evidence from complementary dimensionality analyses, and theoretical/content coherence were considered jointly. Accordingly, an item exceeding one screening threshold was not automatically removed when the remaining psychometric indicators and its conceptual relevance supported retention.

Model fit was evaluated using multiple indices, including *χ*^2^, RMSEA with 90% confidence intervals, Tucker–Lewis Index (TLI), root mean square of residuals (RMSR), and BIC (Browne and Cudeck, 1992; Hu and Bentler, 1999; Schwarz, 1978; Steiger, 1990). Values of RMSEA ≤ 0.06 and RMSR ≤ 0.08 were considered indicative of good fit, although these thresholds were interpreted cautiously given the exploratory nature of the analysis (Hu and Bentler, 1999).

As a sensitivity analysis, the proposed two-factor structure was additionally evaluated by treating the seven CENOAM items as ordered variables and estimating the model using the weighted least squares mean- and variance-adjusted estimator (WLSMV). The model specified Items 2, 3, and 6 as indicators of Familiarity and Items 1, 4, 5, and 7 as indicators of Competence–Demand. This analysis was used to examine whether the proposed item-to-factor allocation remained evident under an ordinal treatment of the response scale.

As complementary analyses of dimensionality, omega hierarchical (*ωh*) and Exploratory Graph Analysis (EGA) were estimated. Omega hierarchical was used to assess the proportion of reliable variance in total scores attributable to a general factor, thereby informing interpretation of the total CENOAM score (Dunn et al., 2014; Zinbarg et al., 2005). EGA was conducted as a network-based approach to evaluate whether the dimensional structure identified through EFA was replicated using a conceptually distinct analytic framework (Golino et al., 2020; Golino and Epskamp, 2017). Structural stability of the EGA solution was examined using parametric bootstrapped EGA (bootEGA) with 1,000 iterations.

Potential redundancy among items was examined using Unique Variable Analysis (UVA), based on weighted topological overlap (wTO) and Pearson correlations. Item pairs with wTO ≥ 0.25 were flagged for further inspection, following current recommendations for redundancy detection in network psychometrics.

#### 2.5.2 Floor and ceiling effects

Floor and ceiling effects were assessed as the percentage of participants scoring the minimum or maximum possible values on the total score. Consistent with established criteria, floor or ceiling effects were considered present when ≥15% of participants achieved the lowest or highest possible score, respectively (Terwee et al., 2007).

#### 2.5.3 Internal consistency

Internal consistency was assessed using Cronbach’s alpha (*α*), McDonald’s omega total (*ω*), and omega hierarchical (*ωh*). Greater interpretative weight was given to omega coefficients, as Cronbach’s alpha assumes tau-equivalence and may yield biased estimates under congeneric measurement conditions (Dunn et al., 2014; McNeish, 2018). In contrast, McDonald’s omega provides a model-based estimate derived from factor loadings and is considered a more accurate representation of reliability (Dunn et al., 2014).

Omega hierarchical (*ωh*) was used to estimate the proportion of variance in total scores attributable to a general factor, independently of group factors. This coefficient is particularly relevant in multidimensional structures, as it allows differentiation between general and specific sources of variance.

To further evaluate the extent to which the CENOAM total score could be interpreted as reflecting a general dimension, explained common variance (ECV) and the percentage of uncontaminated correlations (PUC) were additionally examined. ECV was used to quantify the proportion of common variance attributable to the general factor, whereas PUC quantified the proportion of item correlations influenced only by the general factor. ECV, PUC, and *ωh* were considered jointly when evaluating the strength of the general dimension rather than using any single index as definitive evidence of unidimensionality. An explicit orthogonal bifactor model was also examined as a complementary analysis to evaluate the general and specific components of score variance.

Item-deletion analyses were additionally performed by recalculating *ω* and *ωh* after removing each CENOAM item individually, using the same estimation procedure applied to the complete scale. These analyses were used to examine whether removal of any individual item produced a meaningful improvement in either total reliability or general-factor saturation.

Reliability coefficients were interpreted following conventional guidelines: values ≥0.70 were considered acceptable, ≥0.80 good, and ≥0.90 excellent, whereas values >0.95 were interpreted cautiously due to potential item redundancy (Clark and Watson, 1995; Prinsen et al., 2018; Terwee et al., 2007).

#### 2.5.4 Relation with other variables

Relations with other variables were examined using Pearson correlation coefficients between the CENOAM total score and its two subscale scores (Familiarity and Competence–Demand) and external variables selected on theoretical grounds, including motor imagery ability, physical activity, pain catastrophizing, fear of movement, pain intensity, and pain self-efficacy. These analyses were conducted separately for asymptomatic participants and participants with CLBP, as applicable to the measures available in each group.

Although these hypotheses were not formally prespecified or preregistered before data analysis, the theoretically expected pattern was that higher CENOAM scores would be positively associated with motor imagery ability, physical activity, and pain self-efficacy, and negatively associated with pain catastrophizing and fear of movement. In participants with CLBP, higher pain intensity was additionally expected to be associated with lower CENOAM scores. Because these measures assess theoretically related but non-equivalent constructs, associations were expected to be predominantly weak to moderate rather than uniformly strong.

No adjustment for multiple comparisons was applied because these analyses were intended to examine the pattern, direction, magnitude, and precision of theoretically motivated relationships with external variables rather than to provide confirmatory hypothesis testing. Correlation coefficients were therefore interpreted together with their 95% confidence intervals. Correlation magnitudes were interpreted as negligible (0.00–0.10), weak (0.10–0.39), moderate (0.40–0.69), strong (0.70–0.89), and very strong (0.90–1.00; Schober and Schwarte, 2018).

- Capacity for Generating Motor Imagery. The MIQ-R assesses the capacity for motor imagery (MI) using 8 items, divided into kinesthetic (MIQ-R_K) and visual (MIQ-R_V) subscales. Participants rate the ease with which they can imagine various actions on a scale from 1 (“very difficult”) to 7 (“very easy”). This assessment tool has shown adequate internal consistency in both subscales, as documented by Campos and Gonzalez (2010). Higher scores denote easier imagery.
- Pain Catastrophizing. The Spanish version of the Pain Catastrophizing Scale, a reliable measurement instrument (ICC = 0.84) with adequate internal consistency (Cronbach’s *α* = 0.79), was used to assess the degree of catastrophic thinking related to painful experiences (García Campayo et al., 2008). This scale comprises 13 items organized into three domains: rumination (4 items), which measures persistent worry and inability to inhibit pain-related thoughts; magnification (3 items), which evaluates overestimation of pain unpleasantness; and helplessness (6 items), reflecting the loss of hope regarding relief from detrimental physical and/or psychological aspects (García Campayo et al., 2008).
- Fear of Movement. Fear of movement was evaluated using the Spanish version of the Tampa Scale for Kinesiophobia (TSK-11), which has shown adequate internal consistency (Cronbach’s *α* = 0.81). This instrument consists of 11 items divided into two subscales—fear of physical activity and fear of injury. Each item is rated on a 4-point Likert scale (1 = “strongly disagree,” 2 = “disagree,” 3 = “agree,” 4 = “strongly agree”). Total scores range from 11 to 44 points, with higher scores indicating a greater level of kinesiophobia (Gomez-Perez et al., 2011).
- Physical activity levels were evaluated using the short Spanish version of the International Physical Activity Questionnaire (IPAQ-S). For the analyses of relations with other variables, physical activity was treated as a continuous variable and expressed as total MET-min/week rather than as an ordinal activity category. The IPAQ-S shows test–retest reliability *r* = 0.76 (95% CI 0.73–0.77) and adequate validity *r* = 0.67 (95% CI 0.64–0.70; Mantilla Toloza and Gómez-Conesa, 2007).
- Pain Intensity. Pain intensity was self-reported using a numeric rating scale (0–10/10). A score of 0 corresponds to “no pain,” and a score of 10 represents the “worst possible pain.” This scale has shown excellent test–retest reliability with an ICC range of 0.89 to 0.98 (Sendlbeck et al., 2015).
- Self-Efficacy in Chronic Pain. The level of self-efficacy was assessed using the Spanish version of the Chronic Pain Self-Efficacy Scale (CPSS), which has acceptable psychometric properties for evaluating perceived self-efficacy and the ability to cope with the consequences of chronic pain (Cronbach’s *α* = 0.91; Martín-Aragón et al., 1999). This version comprises 19 items subdivided into three dimensions: self-efficacy for symptom control coping, self-efficacy in pain management, and self-efficacy in physical function. The final score ranges from 0 to 190, and the total score is obtained by summing the three dimensions; higher scores indicate greater self-efficacy (Martín-Aragón et al., 1999). In asymptomatic participants, the CPSS was completed by asking participants to consider their perceived ability to cope with pain-related situations if they were to experience pain or, where applicable, in relation to previous pain experiences. Because the CPSS was originally developed for chronic pain populations, associations involving CPSS scores in asymptomatic participants should be interpreted cautiously.

#### 2.5.5 Discriminant validity

Known-groups validity was examined by comparing participants with CLBP and asymptomatic controls on the CENOAM total and subscale scores. Normality within each group was assessed using Q–Q plots, histograms, and the Shapiro–Wilk test. Homogeneity of variances was examined using Levene’s test. Initial unadjusted comparisons were conducted using Welch’s independent-samples *t* tests. Results are presented as mean differences with 95% CIs and standardized effect sizes using Cohen’s d with 95% CIs, interpreted according to conventional benchmarks: small (0.20), medium (0.50), and large (0.80; Cohen, 1988).

To examine whether the observed between-group differences could be explained by measured demographic characteristics, adjusted linear regression models were fitted separately for the CENOAM total score, Familiarity, and Competence–Demand, with group as the primary predictor and age, sex, BMI, and educational level included as covariates. Heteroscedasticity-consistent HC3 standard errors were used for statistical inference. Adjusted group coefficients are reported with 95% confidence intervals.

## 3 Results

### 3.1 Item development and domain identification

The instrument was developed from a synthesis of the relevant literature together with findings from the qualitative phase of the project. Based on this evidence, a preliminary pool of 11 items was generated. After iterative refinement, 10 items were retained and organized into four hypothesized domains.

An initial working definition of the target construct was formulated to guide item development: the evaluation of participants’ perception, understanding, and response to exercises or movements elicited through action observation.

All items were scored on a 0–10 numerical rating scale, with item-specific anchors designed to maximize interpretability and response discrimination. The initial item pool, domain assignments, and domain definitions are presented in Table 1.

**Table 1:** Initial item pool, conceptual domains, and operational definitions of the CENOAM during the instrument development phase.

Domain	Definition	Item stems (0–10)
Perceived demand	Perceived difficulty and effort required to perform the observed motor action.	(1) *How difficult is the observed movement to perform?* (0 = very easy; 10 = very difficult). (2) *How much physical effort would it require to perform the movement for eight repetitions?* (0 = no effort; 10 = extreme effort)
Familiarity with motor actions	How familiar the movement is and how often it has been performed.	(1) *How familiar is this movement to you?* (0 = not at all familiar; 10 = very familiar). (2) *How frequently have you performed this movement in the past?* (0 = never; 10 = very frequently)
Competence	Perceived capability to execute the movement, clarity about its elements, and interest in practicing it.	(1) *After observing the movement, how capable do you feel of performing it?* (0 = completely unable; 10 = fully capable). (2) *When observing the movement, how clearly can you identify its key elements (start/end position, direction, speed)?* (0 = not clear at all; 10 = extremely clear). (3) *How interested are you in practicing this movement or exercise?* (0 = no interest; 10 = very interested)
Emotional response	Affective reactions (fear, anticipated pain, perceived risk of injury) linked to performing the observed movement.	(1) *To what extent does observing this movement make you afraid to perform it?* (0 = not at all; 10 = extremely). (2) *How painful do you expect performing this movement would be?* (0 = no pain; 10 = extreme pain). (3) *How risky for injury do you consider this movement to be?* (0 = not risky at all; 10 = very risky)

The Emotional Response domain represents items included in the initial item pool during the instrument development phase. As shown in Table 2, these items were subsequently removed following the expert content validity assessment due to insufficient conceptual specificity and applicability across populations.

If you want all scores to point in the same direction (e.g., higher = better), plan to reverse-score the Emotional Response items.

Keep “eight repetitions” only if it matches the tasks; otherwise use “several repetitions” for generality.

### 3.2 Content validity and pilot testing

Content validity was assessed by an expert panel using Aiken’s *V* and 95% confidence intervals for relevance, comprehensiveness, and comprehensibility. The prespecified Aiken’s *V* < 0.70 threshold was applied to the point estimate as a screening indicator rather than as an automatic item-deletion criterion. Confidence intervals were used to characterize the precision of the estimates and were not used to determine item retention.

Three items belonging to the Emotional Response domain were removed based on the combined quantitative and qualitative evaluation. Their Aiken’s *V* point estimates included values below 0.70 for relevance and/or comprehensiveness (Item 1: 0.50 for relevance and 0.56 for comprehensiveness; Item 2: 0.69 for relevance; Item 3: 0.63 for relevance, comprehensiveness, and comprehensibility). In addition, qualitative expert feedback identified concerns regarding ambiguity, conceptual specificity, and applicability across clinical and non-clinical populations.

The remaining seven items showed satisfactory content validity, with Aiken’s *V* point estimates ranging from 0.75 to 0.94 across the three evaluated criteria. Item-level Aiken’s *V* coefficients and their 95% confidence intervals are reported in Table 2.

**Table 2:** Content analysis by expert judges.

Dimensions and items	Relevance Aiken’s *V* (95% CI)	Exhaustiveness Aiken’s *V* (95% CI)	Comprehensibility Aiken’s *V* (95% CI)	Comments
Perceived demand	0.94 (0.72, 0.99)	0.75 (0.51, 0.90)	0.94 (0.72, 0.99)	
Item 1: How would you rate the difficulty of the observed movement?	0.88 (0.64, 0.97)	0.81 (0.57, 0.93)	0.81 (0.57, 0.93)	Judge 8: This item does not account for how perceived difficulty varies greatly depending on the participant’s physical condition.
Item 2: Rate the physical effort it would take to perform the observed movement for 8 repetitions.	0.94 (0.72, 0.99)	0.88 (0.64, 0.97)	0.75 (0.51, 0.90)	Judge 3: The choice of ‘8 repetitions’ is questionable as effort may vary with repetition count.
Familiarity with motor actions	0.81 (0.57, 0.93)	0.88 (0.64, 0.97)	0.81 (0.57, 0.93)	
Item 1: Does the observed movement seem familiar to you?	0.75 (0.51, 0.90)	0.81 (0.57, 0.93)	0.88 (0.64, 0.97)	Judge 3: ‘Familiar’ is ambiguous and may be interpreted as prior experience or visual recognition.
Item 2: Have you previously performed the observed movement?	0.75 (0.51, 0.90)	0.88 (0.64, 0.97)	0.75 (0.51, 0.90)	
Competence	0.81 (0.57, 0.93)	0.75 (0.51, 0.90)	0.94 (0.72, 0.99)	
Item 1: After watching the movement, do you feel capable of performing it?	0.75 (0.51, 0.90)	0.94 (0.72, 0.99)	0.88 (0.64, 0.97)	Judge 5: Perceived capability can be influenced by previous injuries or physical condition.
Item 2: Rate the clarity with which you identified movement components (start/end position, direction, speed).	0.81 (0.57, 0.93)	0.88 (0.64, 0.97)	0.75 (0.51, 0.90)	
Item 3: Does observing this movement spark your interest in practicing it?	0.81 (0.57, 0.93)	0.81 (0.57, 0.93)	0.81 (0.57, 0.93)	
Emotional response	0.75 (0.51, 0.90)	0.63 (0.39, 0.82)	0.63 (0.39, 0.82)	
Item 1: Does observing this movement make you fearful of having to perform it?	0.50 (0.28, 0.72)	0.56 (0.33, 0.77)	0.88 (0.64, 0.97)	Judge 5: The item could be improved by including a specific example or context, such as “fear of performing the movement due to the perceived risk of injury or pain.” Judge 6: Fear is not an essential factor for all participants, especially those with prior experience in similar exercises or movements. Judge 8: It is possible that participants may not know whether to think about physical pain, risk of injury, or simply general anxiety about performing a movement. Judge 1: I do not find this item suitable for an instrument intended to be universal; it measures an affective factor that may have only been experienced by patients with pain.
Item 2: Do you think the observed movement could be painful?	0.69 (0.44, 0.86)	0.75 (0.51, 0.90)	0.88 (0.64, 0.97)	Judge 6: The concept of “painful” is highly subjective and could be interpreted in various ways by different individuals. Some may associate it with acute pain, while others may think of chronic pain. Judge 2: The term “painful” is confusing within this type of interpretive process and could be understood differently by different people. Judge 5: The question does not clarify whether it refers to immediate pain or long-term pain. This could mislead respondents. Furthermore, what would happen if I wanted to use the instrument with a non-clinical population, such as athletes? Judge 7: The item fails to consider that individuals with previous conditions (such as chronic pain) may be more inclined to evaluate the movement as painful, regardless of the actual characteristics of the movement.
Item 3: Do you consider the observed movement dangerous and potentially injurious?	0.63 (0.39, 0.82)	0.63 (0.39, 0.82)	0.63 (0.39, 0.82)	Judge 8: The term “dangerous” is overly broad and may encompass anything from minor risks to serious hazards. This item could have been more specific, for example, by asking whether the movement appears to carry a “high risk of injury.” Judge 3: I believe the question prompts the respondent to consider a scenario that might not even be present in their mind. Judge 4: Without a clear context (such as the participant’s current health status or previous experience with similar movements), the item may be evaluated very variably, which could have lowered its score. Judge 1: Participants with a history of injury or fear of injury may rate this item very differently compared to those who have never experienced an injury, potentially distorting the consistency of responses. Judge 7: The perception of danger may be influenced by factors external to the movement itself, such as unclear instructions or the observer’s prior experience, which could prevent the item from accurately reflecting opinions about what the movement represents.

Aiken’s *V*: An index that measures content validity based on expert judges’ evaluations. Values in parentheses represent the 95% confidence interval (CI).

Relevance, exhaustiveness, and comprehensibility are the dimensions evaluated by the expert judges for each item.

Qualitative observations from the judges are presented in the “Comments” column to complement the quantitative assessment.

The resulting seven-item version of the CENOAM was then piloted in 30 participants (15 asymptomatic and 15 with CLBP). Two items representing Perceived Demand were reverse-scored so that higher total scores consistently reflected a more favorable action-observation appraisal profile.

During pilot testing, participants were exposed to three different motor stimuli (trunk flexion, glute bridge, and squat). Mean (SD) ratings were high for clarity, 8.80 (1.70), comprehensibility, 9.02 (1.03), and relevance, 7.33 (2.14). The cognitive-interviewing and pilot-testing stage identified very few recurrent difficulties in understanding the individual CENOAM items. No specific item showed a consistent pattern of major comprehension problems, and no substantial item wording changes were required.

The main recurrent difficulty concerned the administration of multiple movement stimuli. Some participants reported difficulty providing a single global appraisal because their perceptions differed according to the movement being observed. This indicated that CENOAM responses could depend on the specific observed action and that a single judgment across multiple movements could introduce ambiguity. Consequently, the administration protocol for the main psychometric study was refined so that CENOAM responses referred to a single standardized motor stimulus.

### 3.3 Sample characteristics and item descriptives

The psychometric validation sample comprised 280 participants, including 140 asymptomatic individuals (AP) and 140 participants with chronic low back pain (CLBP). Sociodemographic characteristics are presented in Table 3. No statistically significant differences were observed between groups for age (*p* = 0.062). In contrast, participants with CLBP showed a higher body mass index compared to asymptomatic individuals (*p* < 0.001). A statistically significant difference was also observed in sex distribution between groups (*p* = 0.038), whereas educational level showed a borderline association with group membership (*p* = 0.050).

**Table 3:** Descriptive statistics of the sociodemographic characteristics of the sample.

Measure	Asymptomatic (*n* = 140)	CLBP (*n* = 140)	*p*-value	Effect size
Age (years), mean ± SD	36.17 ± 15.00	39.46 ± 14.38	0.062	*d* = 0.22
Sex—Male, *n* (%)	34 (24.3)	51 (36.4)	0.038*	*φ* = 0.12
Sex—Female, *n* (%)	106 (75.7)	89 (63.6)	–	–
BMI (kg/m^2^), mean ± SD	21.25 ± 2.89	25.24 ± 4.46	<0.001***	*d* = 1.06
Education—Vocational, *n* (%)	9 (6.4)	17 (12.1)	0.050	*V* = 0.15
Education—Secondary, *n* (%)	67 (47.9)	49 (35.0)	–	–
Education—Higher, *n* (%)	64 (45.7)	74 (52.9)	–	–

CLBP = chronic low back pain; BMI = body mass index; ^*^p < 0.05, ^***^p < 0.001; *d* = Cohen’s *d*; *φ* = phi coefficient; *V* = Cramer’s *V*.

The CENOAM showed a 100% completion rate, with no missing responses. Item-level descriptive statistics are presented in Table 4, including mean, standard deviation, skewness, kurtosis, factor loadings, communalities, uniqueness values, factorial complexity, and reliability indices under item-deletion conditions.

**Table 4:** Descriptive statistics, factor loadings, and reliability indices of the CENOAM items.

Item	Mean	SD	Skewness	Kurtosis	F1	F2	*h* ^2^	*u* ^2^	Complexity
Item 2	6.25	1.71	0.33	−0.30	**0.92**	0.10	0.85	0.15	1.00
Item 3	6.05	1.55	0.28	−0.36	**0.92**	0.08	0.81	0.19	1.00
Item 6	6.11	1.78	0.14	−0.42	**0.86**	0.15	0.80	0.20	1.01
Item 1	5.72	2.02	−0.50	−0.46	0.22	**0.79**	0.55	0.45	1.04
Item 7	6.31	2.06	−0.60	−0.64	0.15	**0.75**	0.57	0.43	1.00
Item 4	5.93	2.15	−0.20	−0.90	0.12	**0.68**	0.43	0.57	1.02
Item 5	5.93	2.18	0.15	−1.02	0.38	**0.64**	0.77	0.23	1.63

F1 = Familiarity; F2 = Competence/Perceived Demand; *h*^2^ = communality; *u*^2^ = uniqueness. Exploratory factor analysis was conducted using minimum residual (MINRES) extraction with oblimin rotation. Primary loadings are shown in bold.

Item means ranged from 5.72 to 6.31, and standard deviations ranged from 1.55 to 2.18. Skewness values ranged from −0.60 to 0.33, and kurtosis values did not indicate substantial deviations from normality. Floor and ceiling effects were negligible across items and dimensions.

### 3.4 Dimensionality and internal structure

#### 3.4.1 Factorability and sampling adequacy

Sampling adequacy for factor analysis was good, with a Kaiser–Meyer–Olkin (KMO) index of 0.82. Item-level measures of sampling adequacy ranged from 0.79 to 0.87 and are reported in Table 5. Bartlett’s test of sphericity was statistically significant, *χ*^2^(21) = 1,239.44, *p* < 0.001, supporting the factorability of the inter-item correlation matrix.

**Table 5:** Item-level sampling adequacy and item-deleted reliability diagnostics for the CENOAM.

Item	Content	MSA	*ω* if item removed	*ωh* if item removed
Item 1	How would you rate the difficulty of the observed movement? ¿Cómo calificaría la dificultad del movimiento observado?	0.79	0.926	0.593
Item 2	Does the observed movement feel familiar to you? ¿Le resulta familiar el movimiento observado?	0.79	0.905	0.587
Item 3	Have you performed the observed movement before? ¿Ha realizado anteriormente el movimiento observado?	0.80	0.916	0.704
Item 4	After observing the movement, do you feel capable of performing it? ¿Después de observar el movimiento, se siente capaz de realizarlo?	0.80	0.934	0.688
Item 5	While observing the movement, rate how clearly you identified its components (initial/final position, direction, speed) Al observar el movimiento, califique la claridad con la que identificó los elementos (posición inicial/final, dirección, velocidad)	0.87	0.908	0.656
Item 6	Does observing this movement increase your interest in practicing the activity or exercise? ¿La observación de este movimiento despierta su interés en practicar esa actividad o ejercicio?	0.85	0.927	0.670
Item 7	Evaluate the effort required to physically perform the observed movement for eight repetitions Evalúe el esfuerzo que le supondría realizar físicamente el movimiento durante ocho repeticiones	0.84	0.922	0.722

MSA = Measure of Sampling Adequacy. *ω* = McDonald’s omega total. *ωh* = hierarchical omega. Values are computed after removing each item from the total scale. All items showed adequate sampling adequacy (MSA ≥ 0.79). Removal of any item did not result in meaningful improvements in internal consistency.

#### 3.4.2 Dimensionality assessment

A multimethod approach was used to determine the number of latent dimensions. Horn’s parallel analysis indicated a two-factor solution, as only the first two observed eigenvalues exceeded those derived from simulated data (Figure 1A). Inspection of the scree plot showed an inflection after the second factor.

**Figure 1:**
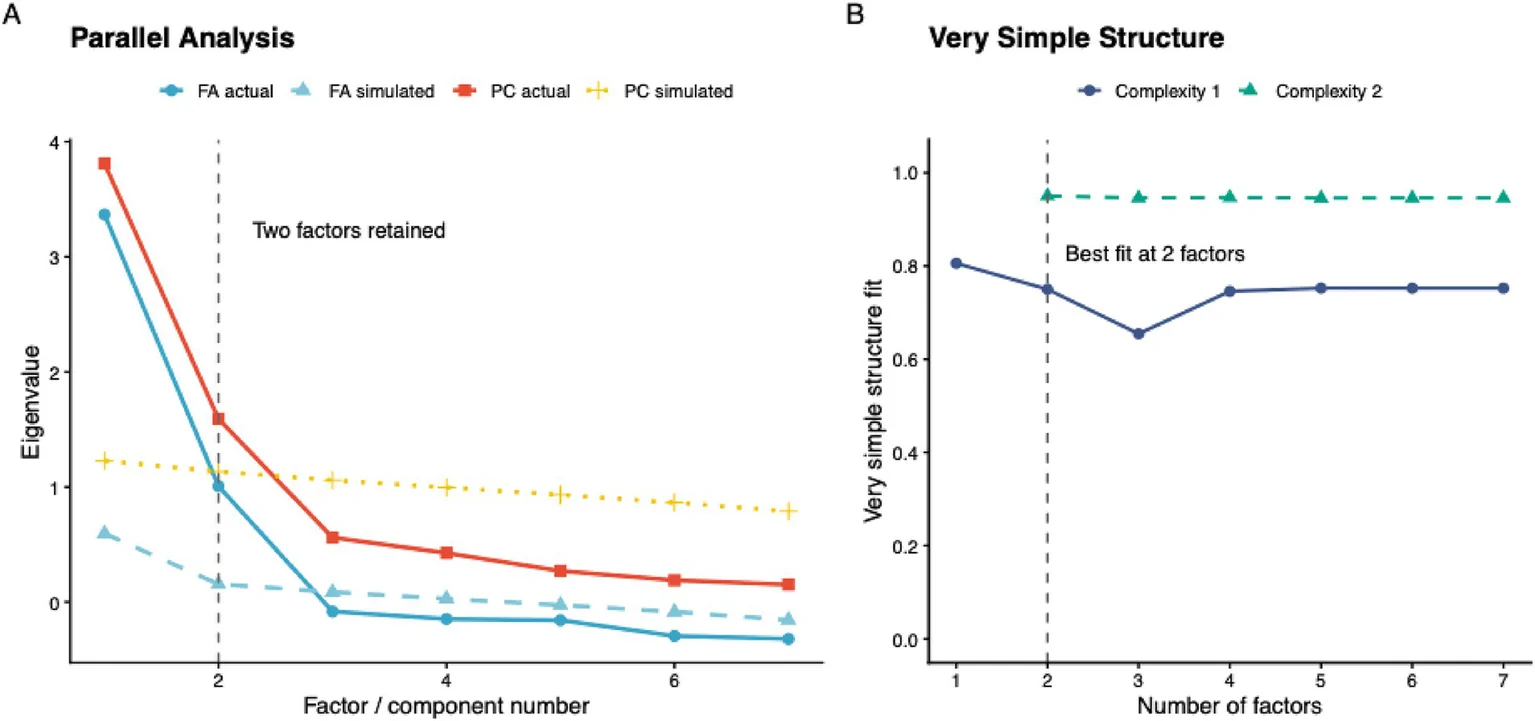
Dimensionality assessment using parallel analysis and Very Simple Structure (VSS). **Panel A** shows Horn’s parallel analysis. Observed eigenvalues derived from factor analysis (FA actual) and principal components (PC actual) are compared against eigenvalues obtained from randomly generated datasets with the same sample size and number of variables (FA simulated and PC simulated). The number of retained factors corresponds to those with observed eigenvalues exceeding simulated values. **Panel B** presents Very Simple Structure (VSS) fit indices across different factor solutions. Complexity 1 and complexity 2 represent different levels of item–factor loading simplification. The highest fit was observed for the two-factor solution.

Velicer’s minimum average partial (MAP) test reached its minimum with two factors (MAP = 0.08). Very Simple Structure (VSS) analysis also supported this solution, with the highest fit observed for two factors (VSS complexity 2 = 0.84; Figure 1B).

Bayesian Information Criterion (BIC) reached its minimum with two factors (BIC = −20.03), whereas the sample-size adjusted BIC suggested a three-factor solution (SABIC = −0.66).

Exploratory Graph Analysis (EGA) identified a two-community structure (Figure 2), grouping items c2, c3, and c6 within one dimension and items c1, c4, c5, and c7 within a second dimension. Bootstrapped EGA (bootEGA) showed that the two-dimensional solution was replicated in 100% of bootstrap samples (median dimensions = 2, 95% CI [2, 2]).

**Figure 2:**
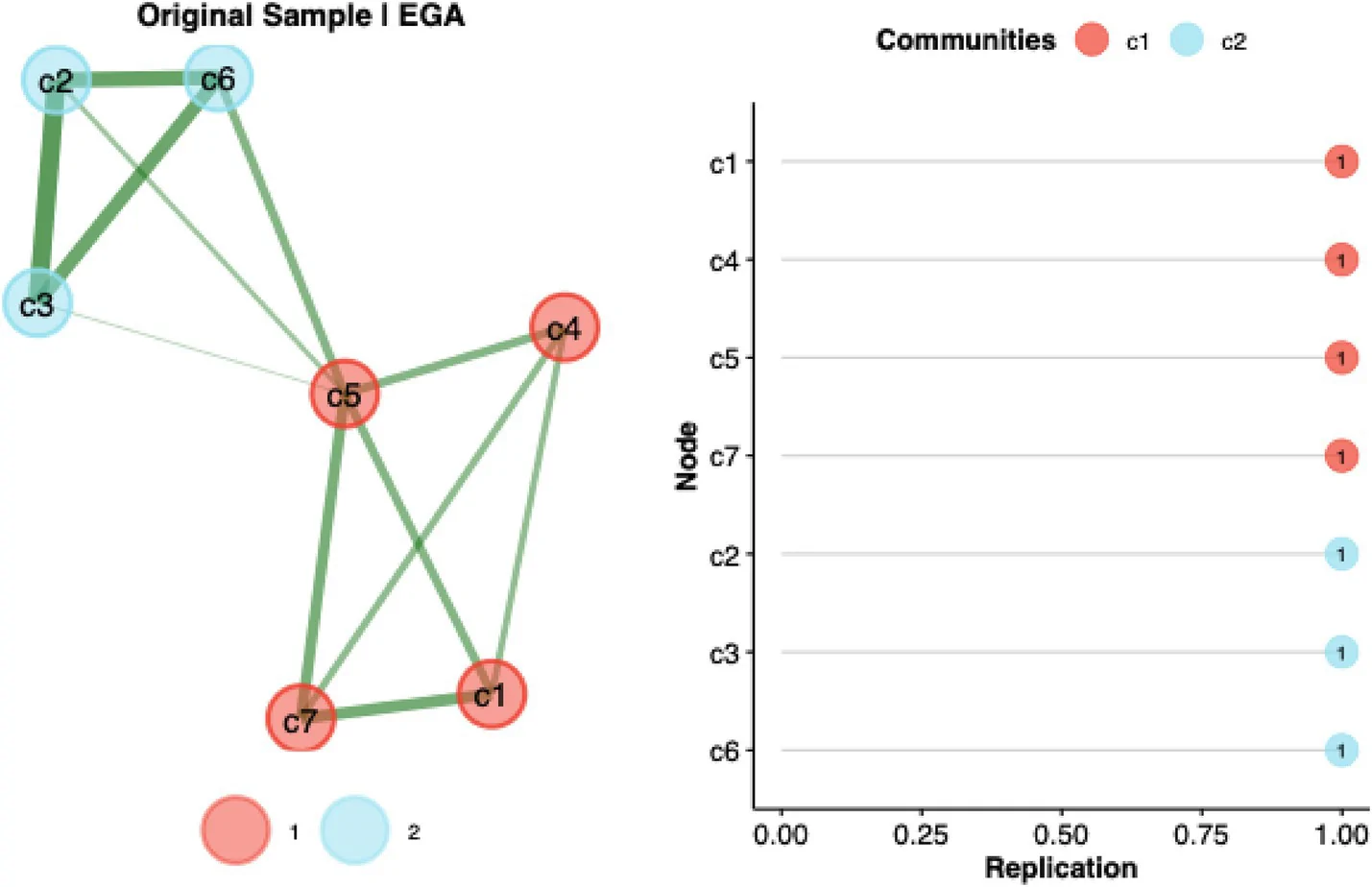
Exploratory Graph Analysis (EGA) network and community structure. Network estimated using a Gaussian Graphical Model with EBICglasso regularization (*γ* = 0.5) and visualized with the Fruchterman–Reingold algorithm. Nodes represent items and edges represent partial correlations. Two communities were identified using the Walktrap algorithm: Familiarity (c2, c3, c6) and Competence and Demand (c1, c4, c5, c7). Edge thickness reflects the strength of the association.

#### 3.4.3 Exploratory factor analysis

Exploratory factor analysis using the minimum residual (MINRES) method with oblimin rotation was conducted for both two- and three-factor solutions. Detailed factor loadings and associated item-level indicators are reported in Table 4.

The two-factor EFA showed mixed model-fit evidence: *χ*^2^(8) = 25.05, *p* = 0.00152, RMSR = 0.020, TLI = 0.963, RMSEA = 0.087 (90% CI [0.050, 0.127]), and BIC = −20.03. Thus, RMSR and TLI were favorable, whereas RMSEA exceeded the prespecified ≤0.06 criterion. The model accounted for 66.8% of the total variance. Items 2, 3, and 6 loaded primarily on Familiarity, whereas Items 1, 4, 5, and 7 loaded primarily on Competence–Demand. The two factors were moderately correlated (*r* = 0.428). Complete pattern and structure matrices are provided in Supplementary Table S4.

Item 5 showed some factorial complexity, with a primary loading of 0.642 on Competence–Demand and a secondary loading of 0.383 on Familiarity. Although the secondary loading exceeded the prespecified 0.30 screening threshold, the primary-to-secondary loading difference was 0.259, exceeding the 0.20 differentiation criterion, and the item showed a high communality (*h*^2^ = 0.77). Because the cross-loading threshold was used as a screening rather than an automatic exclusion criterion, and the remaining psychometric indicators together with the theoretical/content coherence of the item supported its assignment to Competence–Demand, Item 5 was retained.

The three-factor model showed improved absolute fit (RMSR = 0.01; RMSEA = 0.067), but worse parsimony (BIC = −10.18) and increased item complexity, particularly for item c5.

In an ordinal sensitivity analysis using WLSMV, the proposed item-to-factor allocation was reproduced. Standardized loadings were 0.932, 0.910, and 0.909 for Items 2, 3, and 6 on Familiarity, respectively, and 0.633, 0.578, 0.966, and 0.690 for Items 1, 4, 5, and 7 on Competence–Demand, respectively (all *p* < 0.001). The factors correlated at *r* = 0.601 (*p* < 0.001). Global fit evidence was again mixed: scaled *χ*^2^(13) = 85.47, *p* < 0.001, scaled CFI = 0.991, scaled TLI = 0.985, scaled RMSEA = 0.141 (90% CI [0.114, 0.171]), and SRMR = 0.071. Thus, the ordinal analysis reproduced the proposed item-to-factor allocation but did not provide uniformly favorable evidence of global model fit.

#### 3.4.4 Redundancy analysis

Potential item redundancy was examined using Unique Variable Analysis (UVA). Results are presented in Table 6.

**Table 6:** Redundancy analysis of CENOAM items.

Item pair	Pearson *r*	wTO	Redundancy level	Rationale for retention	Decision
Item2–Item3	0.83	0.423	Large to very large	Both reflect familiarity and prior experience with the movement, but distinguish between recognition and execution history	Retained
Item2–Item6	0.82	0.355	Large to very large	Both capture engagement with the observed movement, but represent familiarity and motivational interest	Retained
Item3–Item6	0.80	0.346	Large to very large	Related aspects of motor experience, but differentiate past performance and current interest	Retained
Item1–Item7	0.57	0.272	Moderate to large	Both relate to perceived task demand, but reflect difficulty versus anticipated effort	Retained
Item5–Item7	0.60	0.250	Moderate to large	Both involve appraisal of movement, but distinguish clarity from effort	Retained
Item1–Item5	0.56	0.224	Small to moderate	Both relate to task evaluation, but represent difficulty versus perceptual clarity	Retained
Item4–Item5	0.53	0.221	Small to moderate	Both involve cognitive appraisal, but reflect ability versus perceptual processing	Retained

Pearson correlations (*r*) and weighted topological overlap (wTO) were used to evaluate potential redundancy among items. The wTO index reflects the extent to which two items share similar connectivity patterns within the network structure, with higher values indicating greater redundancy. A threshold of wTO ≥ 0.25 was considered indicative of potential redundancy. Redundancy levels were interpreted as follows: small to moderate (0.10–0.25), moderate to large (0.25–0.35), and large to very large (>0.35). Item pairs exceeding the threshold were examined qualitatively to determine conceptual overlap and distinctiveness. All items were retained based on theoretical relevance and non-equivalence of content despite statistical overlap.

Several item pairs exceeded the predefined redundancy threshold (wTO ≥ 0.25), including c2–c3 (wTO = 0.42), c2–c6 (wTO = 0.36), c3–c6 (wTO = 0.35), c1–c7 (wTO = 0.27), and c5–c7 (wTO = 0.25).

#### 3.4.5 Reliability and general factor saturation

Reliability analyses indicated high internal consistency for the total score (*α* = 0.85; *ω* = 0.915) and for the Familiarity (*α* = 0.93; *ω* = 0.93) and Competence–Demand (*α* = 0.82; *ω* = 0.82) subscales. Importantly, omega total and omega hierarchical quantify different components of score variance. Whereas omega total reflects reliable variance attributable to both general and specific common factors, omega hierarchical estimates the proportion of total-score variance attributable specifically to the general component after separating specific-factor variance. In the present data, *ωh* was 0.548, indicating a meaningful general component while simultaneously confirming that substantial variance remained associated with the specific dimensions. The omega hierarchical structure is illustrated in Figure 3.

**Figure 3:**
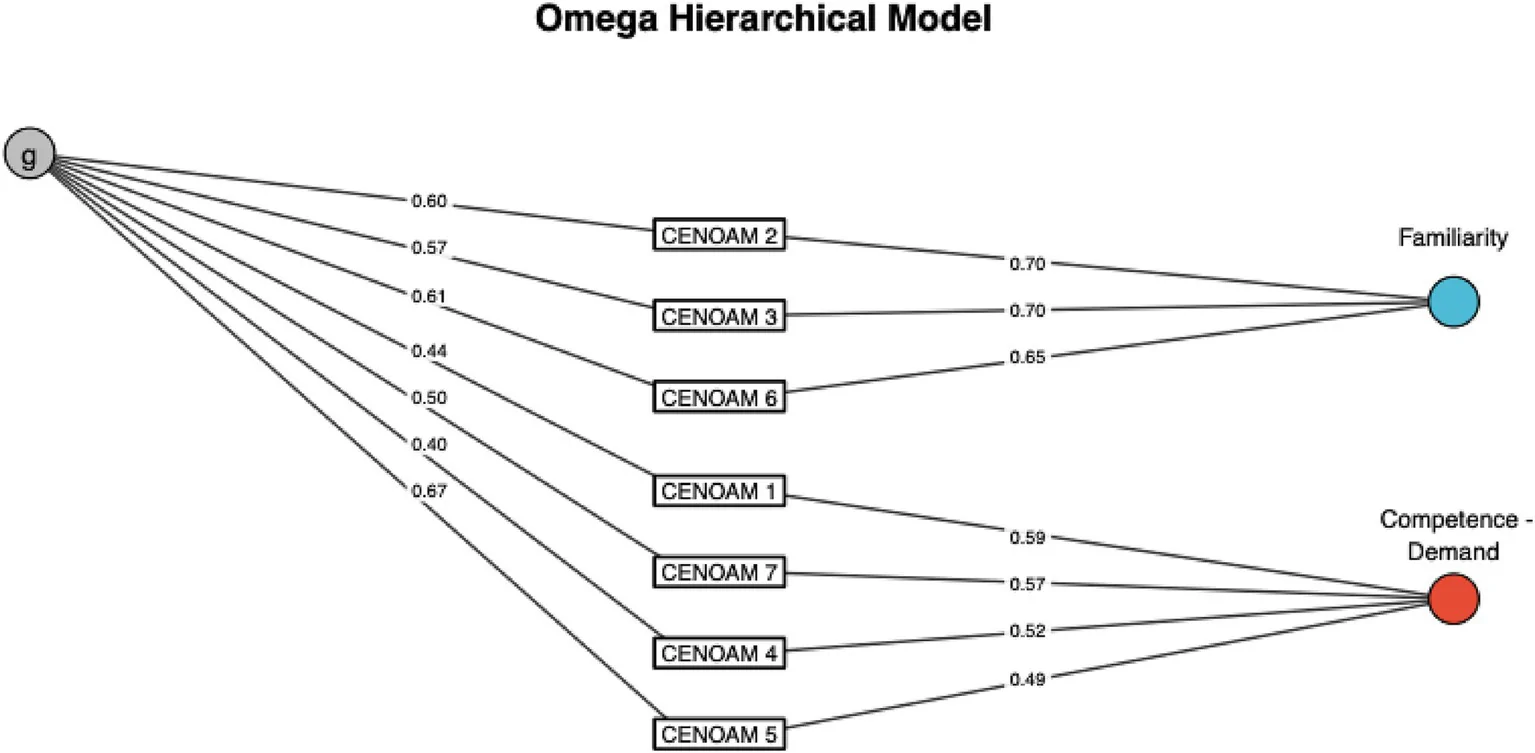
Omega hierarchical model of the CENOAM scale. Schmid–Leiman solution illustrating the general factor (g) and group-specific factors. Loadings represent standardized coefficients. The model illustrates the contribution of both general and specific dimensions to item variance.

Additional analyses yielded an explained common variance (ECV) of 0.441 and a percentage of uncontaminated correlations (PUC) of 0.571. These findings do not support a strictly unidimensional interpretation of CENOAM, but are compatible with a multidimensional structure containing both a general component and meaningful specific dimensions. An explicit orthogonal bifactor model was also examined but produced an inadmissible Heywood solution and therefore could not be meaningfully interpreted.

Item-deletion analyses using the same procedure that reproduced the full-scale omega coefficients showed no substantial improvement in either omega total or omega hierarchical after deletion of any item (Table 5). Accordingly, Familiarity and Competence–Demand should retain interpretative priority when dimension-specific information is required, whereas the total score may be retained as a complementary broader composite of movement appraisal and should not be interpreted as a purely unidimensional score.

#### 3.4.6 Floor and ceiling effects

Floor and ceiling effects for the total score were 0.36 and 0.36%, respectively. For Familiarity, floor and ceiling effects were 1.79 and 2.14%. For Competence and Demand, both were 0.36%.

#### 3.4.7 Relations with external variables

Group-specific analyses showed differences in the relationships among CENOAM scores. In asymptomatic participants, the correlations were *r* = 0.598 between Total and Familiarity, *r* = 0.488 between Total and Competence–Demand, and *r* = 0.334 between Familiarity and Competence–Demand. In participants with CLBP, the corresponding correlations were *r* = 0.609, *r* = 0.806, and *r* = 0.696, respectively. Complete item-level correlation matrices for both groups are provided in the Supplementary material.

Updated Pearson correlation matrices for asymptomatic participants and participants with CLBP are presented separately in Figure 4 (panels A,B). Complete correlation estimates for the CENOAM total score, Familiarity, and Competence–Demand, including 95% confidence intervals and *p*-values, are provided in Supplementary Table S5.

**Figure 4:**
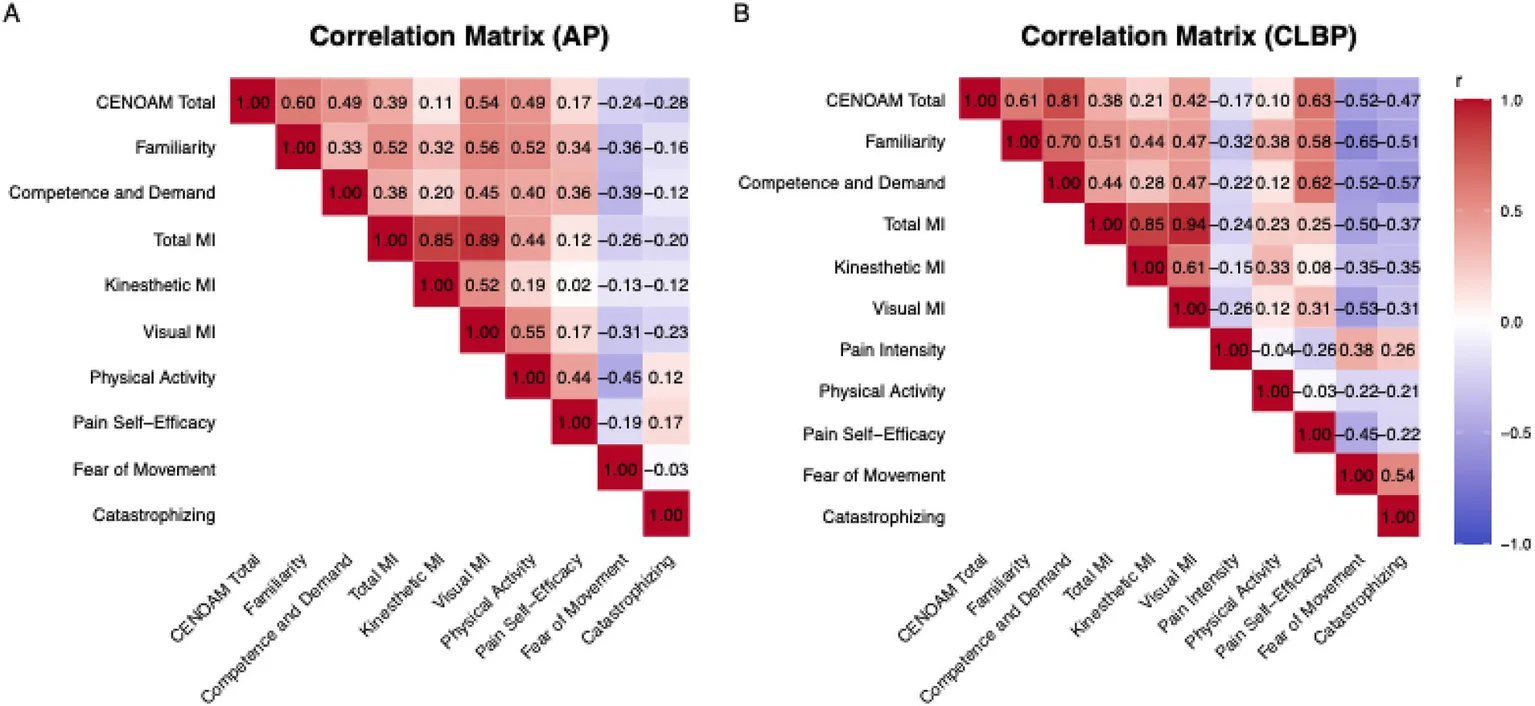
Correlation matrices showing relations between CENOAM scores and external variables in asymptomatic participants and participants with CLBP. **Panel A** shows Pearson correlations for asymptomatic participants, and **Panel B** for participants with CLBP. Color intensity represents the magnitude and direction of associations. CENOAM scores are displayed alongside external variables, including physical activity, motor imagery, fear of movement, pain catastrophizing, and pain self-efficacy.

Across the 45 examined associations, all point estimates were in the theoretically expected direction, and 39 of 45 (86.7%) had 95% confidence intervals excluding zero.

In asymptomatic participants, the CENOAM total score showed moderate positive relationships with visual motor imagery (*r* = 0.536, 95% CI [0.407, 0.645]) and physical activity (*r* = 0.485, 95% CI [0.347, 0.603]), and a weak positive relationship with total motor imagery (*r* = 0.391, 95% CI [0.240, 0.523]). Negative relationships were observed with pain catastrophizing (*r* = −0.282, 95% CI [−0.428, −0.122]) and fear of movement (*r* = −0.242, 95% CI [−0.392, −0.079]).

In participants with CLBP, the CENOAM total score showed positive relationships with pain self-efficacy (*r* = 0.626, 95% CI [0.513, 0.717]), visual motor imagery (*r* = 0.422, 95% CI [0.276, 0.550]), total motor imagery (*r* = 0.377, 95% CI [0.225, 0.511]), and kinesthetic motor imagery (*r* = 0.209, 95% CI [0.045, 0.363]). Negative relationships were observed with fear of movement (*r* = −0.517, 95% CI [−0.629, −0.384]), pain catastrophizing (*r* = −0.466, 95% CI [−0.586, −0.325]), and pain intensity (*r* = −0.167, 95% CI [−0.324, −0.001]). In contrast, the relationship between the CENOAM total score and physical activity was negligible and imprecisely estimated (*r* = 0.098, 95% CI [−0.069, 0.259]).

Overall, the observed relationships generally supported the theoretical directional expectations, although their magnitude and consistency varied across CENOAM dimensions and groups. Physical activity showed the least consistent pattern, particularly in participants with CLBP.

#### 3.4.8 Known-groups (discriminant) validity

Inspection of Q–Q plots and histograms indicated deviations from normality across groups. Shapiro–Wilk tests were statistically significant (all *p* < 0.05). Levene’s test indicated heterogeneity of variances for Familiarity and Competence–Demand (*p* < 0.01) and homogeneity for the total score (*p* = 0.234).

Welch’s *t*-tests showed that participants with chronic low back pain scored significantly lower than asymptomatic participants on all CENOAM dimensions (all *p* < 0.001).

Mean differences were:

- CENOAM Total: MD = 16.66, 95% CI [15.44, 17.89], *t*(277.88) = 26.85.
- Competence–Demand: MD = 9.91, 95% CI [8.81, 11.00], *t*(219.66) = 17.83.
- Familiarity: MD = 6.76, 95% CI [5.98, 7.53], *t*(265.18) = 17.18.

Standardized effect sizes (Cohen’s *d*) ranged from 2.05 to 3.21, and Cliff’s delta ranged from 0.86 to 0.97.

Group score distributions for all CENOAM dimensions are shown in Figure 5.

**Figure 5:**
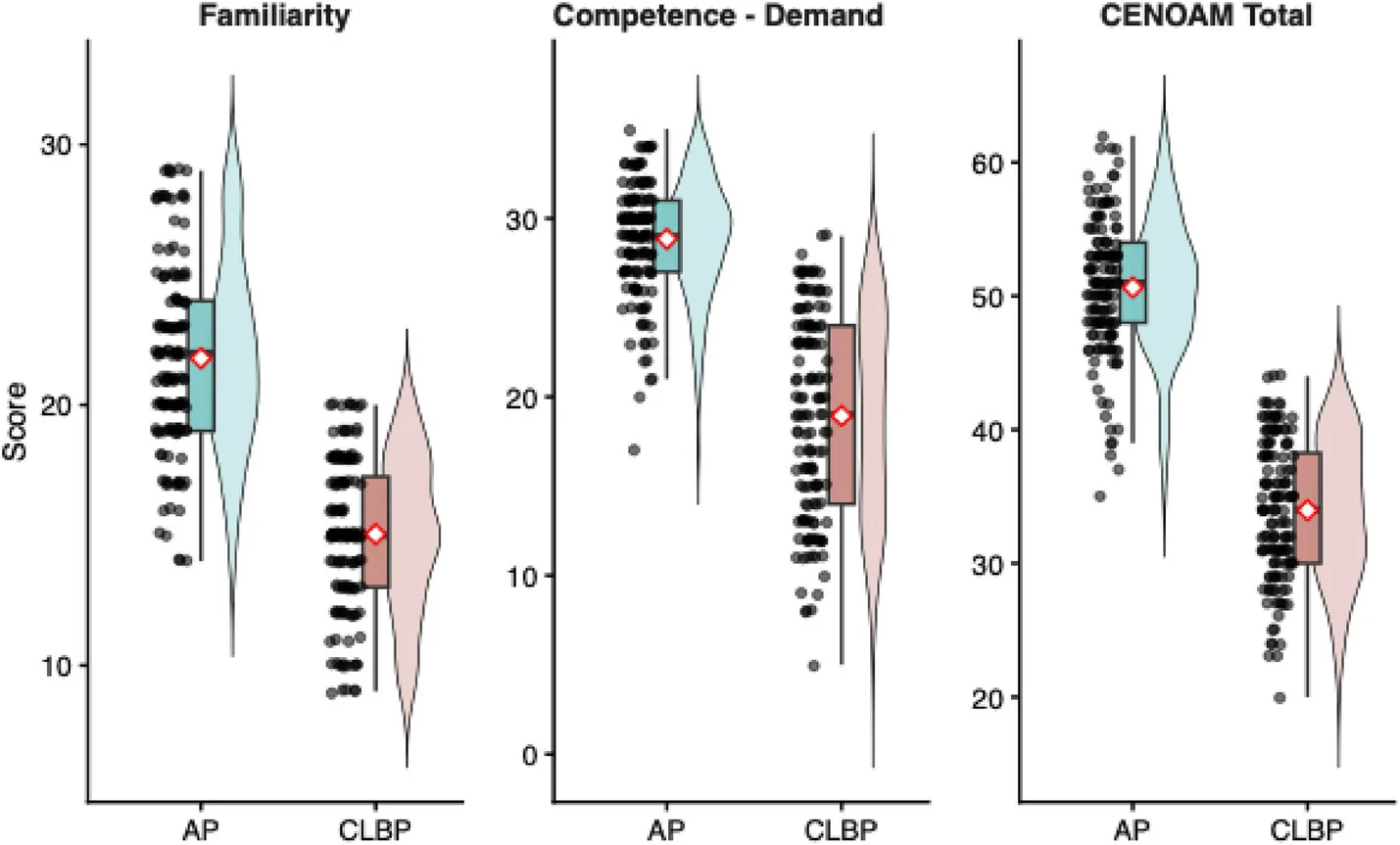
Distribution of CENOAM scores across asymptomatic and CLBP groups. Violin plots, boxplots, and individual data points are shown for Familiarity, Competence-Demand, and CENOAM Total in asymptomatic participants (AP) and participants with CLBP. White diamonds indicate group means. Distributions illustrate lower scores in the CLBP group across all dimensions.

## 4 Discussion

This study developed the CENOAM and provided initial evidence supporting the validity of its scores as a measure of how individuals appraise observed movements. Evidence derived from content validation, exploratory factor analysis, network-based dimensionality methods, reliability estimation, convergent patterns, and known-groups comparisons consistently supported a parsimonious two-dimensional structure, comprising Familiarity and Competence–Demand. Across a sample of 280 participants (140 with CLBP and 140 asymptomatic individuals), the instrument demonstrated adequate distributional properties, absence of floor and ceiling effects, high internal consistency, and strong discriminative capacity between clinical and non-clinical groups. Overall, these findings are aligned with recommended methodological standards for the development and evaluation of patient-reported outcome measures (Reeve et al., 2013).

### 4.1 Construct representation in light of prior theory

Content validation indicated that the retained items were relevant, comprehensive, and clearly interpretable, in accordance with current recommendations for instrument development (Artino et al., 2014; Mokkink et al., 2010a, b; Prinsen et al., 2018). However, despite the initial theoretical framework proposing a more differentiated structure, the empirical findings consistently supported a more parsimonious two-factor solution across complementary approaches, including parallel analysis, MAP, VSS, BIC, and EGA, with full replication in bootstrapped analyses. This convergence across methods strengthens confidence in the identified dimensional structure.

The retained dimensions capture two complementary components of movement appraisal during action observation. Familiarity reflects prior experience, recognition, and exposure to the observed movement, processes known to facilitate action understanding and motor resonance (Calvo-Merino et al., 2006; Gardner et al., 2015). In contrast, Competence–Demand integrates perceived capability, perceived clarity of the observed action, and anticipated effort or difficulty. Importantly, this empirical integration suggests that, at the subjective level, individuals do not clearly differentiate between feeling capable of performing a movement and perceiving it as effortful. Instead, these elements appear to converge into a unified judgment of action feasibility. These evaluations, however, represent subjective appraisals elicited by the observed movement and should not be interpreted as direct indicators of the individual’s actual motor capacity or objective ability to execute the action.

This finding is theoretically coherent with contemporary models of motor cognition, which conceptualize action observation as involving not only sensorimotor simulation but also higher-order inferential and evaluative processes (Abreu et al., 2023; Brass et al., 2007). From this perspective, appraisal of an observed movement is not purely representational but also incorporates cost–benefit estimations, anticipated effort, and self-efficacy beliefs. The moderate correlation observed between dimensions further supports a structure in which familiarity and evaluative judgments are related but non-redundant components contributing to action understanding.

### 4.2 General factor and score interpretation

The dimensionality analyses generally converged on a parsimonious two-factor organization comprising Familiarity and Competence–Demand, although the strength of evidence differed across analytical criteria. Parallel analysis, MAP, VSS, BIC, and EGA favored a two-dimensional solution, and the primary EFA loadings were largely consistent with this organization. However, global model-fit evidence was mixed: RMSR and TLI were favorable, whereas RMSEA exceeded the prespecified criterion. An ordinal WLSMV sensitivity analysis reproduced the proposed item-to-factor allocation but similarly yielded mixed global fit indices. Item 5 also showed moderate factorial complexity in the exploratory solution. Accordingly, the present findings should be interpreted as initial evidence supporting a parsimonious two-dimensional organization rather than as demonstrating an unequivocally robust or definitive factorial structure. Further independent confirmatory evaluation is warranted.

The reliability and bifactor findings suggest that CENOAM scores contain both a meaningful common component and substantial dimension-specific variance. Although the total score demonstrated high internal consistency, the omega hierarchical indicated that only part of the reliable variance could be attributed to a general factor. This interpretation was further supported by the ECV (0.441) and PUC (0.571) values, which did not support a strict unidimensional representation of the instrument. Moreover, the attempted orthogonal bifactor model resulted in an inadmissible Heywood case, preventing a stable decomposition of variance into a single general factor and orthogonal specific factors. Taken together, these findings suggest that Familiarity and Competence–Demand represent meaningful dimensions that coexist with, rather than are fully explained by, an overarching movement-appraisal factor.

From a practical perspective, the results support retaining both subscale scores and the total score, albeit for different purposes. The Familiarity and Competence–Demand dimensions appear to capture distinct and potentially informative aspects of movement appraisal and therefore should receive primary emphasis when dimension-specific interpretation is required. At the same time, the presence of a meaningful general component justifies the use of an overall score as a broader indicator of movement appraisal. However, this total score should be interpreted as a composite reflecting both shared and dimension-specific influences rather than as evidence of a purely unidimensional construct. Consistent with this interpretation, item-deletion analyses showed no meaningful improvement in either omega total or omega hierarchical, suggesting that the proposed score structure is reasonably stable and is not dependent on any single item.

### 4.3 Relations with external constructs

Relations with external variables generally followed the theoretically expected directions, although their magnitude and consistency varied across CENOAM dimensions and between groups. Motor imagery showed predominantly positive associations with CENOAM scores, supporting the expected relationship between movement appraisal and representational aspects of motor cognition. In participants with CLBP, pain self-efficacy showed moderate positive associations with CENOAM scores, whereas pain catastrophizing and fear of movement showed moderate negative associations. Pain intensity was also negatively related to CENOAM scores, although these associations were generally weak.

Physical activity showed a less consistent pattern. Positive associations were observed in asymptomatic participants and with Familiarity in participants with CLBP, whereas relationships with the CENOAM total score and Competence–Demand were negligible or weak and imprecisely estimated in the CLBP group. Thus, the findings provide general, but not uniform, support for the theoretical expectations concerning relations with other variables.

These findings should be interpreted as evidence that CENOAM scores are related to, but not interchangeable with, established cognitive, affective, and behavioral constructs. Because multiple correlations were examined without adjustment for multiplicity, interpretation should emphasize the overall pattern, magnitude, and precision of the associations rather than isolated statistical significance. In addition, associations involving the CPSS in asymptomatic participants should be interpreted cautiously because this instrument was developed specifically to assess self-efficacy in the context of chronic pain.

These relationships can also be interpreted within the framework of Social Learning Theory. Bandura’s account of observational learning proposes that exposure to an observed action is not sufficient, by itself, to determine subsequent learning or behavior. Rather, observational learning involves several interrelated processes, including attention to the modeled action, retention of relevant information, the capacity for motor reproduction, motivation, and self-efficacy beliefs regarding one’s ability to perform the behavior (Bandura and Jeffrey, 1973; Bandura and Walters, 1977). From this perspective, the way an individual subjectively appraises an observed movement may represent an important component of the cognitive processing that occurs during action observation.

The dimensions identified in CENOAM can be considered in relation to these processes. Familiarity may reflect previous exposure to and recognition of the observed action, which may facilitate the interpretation and representation of the movement. Competence–Demand, in contrast, reflects the individual’s subjective appraisal of the feasibility, clarity, difficulty, and anticipated effort associated with the observed action. These appraisals are conceptually related to efficacy beliefs and to the evaluation of whether an observed behavior is perceived as potentially reproducible by the observer. In this context, the positive associations observed between CENOAM scores and pain self-efficacy in participants with CLBP are theoretically coherent with the proposition that efficacy beliefs influence how individuals evaluate their capacity to engage with behavior (Bandura, 1977; Chong et al., 2009).

The positive associations between CENOAM scores and motor imagery ability should nevertheless be interpreted at the level of subjective cognitive-motor appraisal. CENOAM assesses how an individual perceives and evaluates an observed movement; it does not objectively assess the individual’s capacity to execute that movement. Similarly, self-reported motor imagery ability and objective motor performance should not be assumed to be equivalent. Evidence from research examining motor imagery and physical performance suggests that perceived motor imagery ability and objective functional performance may diverge (Çiftçi and Yılmaz, 2024). Therefore, the present associations indicate convergence between related subjective aspects of movement representation and appraisal, but they do not demonstrate that more favorable CENOAM scores correspond to superior actual motor performance.

Importantly, however, CENOAM should not be interpreted as a measure of observational learning, motor learning, or actual motor capacity. It assesses the subjective appraisal elicited by a specific observed movement. Likewise, favorable appraisal of an observed action does not necessarily imply that the individual can execute that action successfully or that observation will result in subsequent behavioral engagement. The present cross-sectional associations therefore support a theoretical relationship between movement appraisal, motor representation, and self-efficacy, but they do not establish a causal pathway from favorable appraisal to motor performance, adherence, or functional improvement.

The substantial differences in CENOAM scores observed between participants with CLBP and asymptomatic participants should be interpreted at the observed-score level. The present study was designed as an initial development and psychometric evaluation of CENOAM rather than as a formal multigroup validation study, and measurement invariance across CLBP and asymptomatic populations has not yet been established. Consequently, the present known-groups findings should not be interpreted as demonstrating equivalent differences in latent construct means between populations. Rather, they provide evidence of substantial differences in observed CENOAM scores whose latent-level comparability requires confirmation in a specifically designed measurement-invariance study.

### 4.4 Known-groups differences and clinical interpretation

Large observed differences in CENOAM scores were identified between participants with CLBP and asymptomatic participants. These differences remained substantial after adjustment for age, sex, BMI, and educational level, with only limited attenuation of the observed score differences after adjustment for these measured demographic characteristics. Nevertheless, these findings should not be interpreted as demonstrating that CLBP status itself causes lower CENOAM scores. The groups were recruited from different settings, residual confounding by unmeasured characteristics remains possible, and direct interpretation of these observed score differences as equivalent latent construct differences requires adequate measurement invariance across groups. Accordingly, the present findings are interpreted as substantial observed between-group score differences rather than definitive evidence of latent construct differences attributable to CLBP.

The direction of these observed differences is consistent with previous research describing alterations in the processing and appraisal of action-related information in chronic pain, including pain-related threat processing, perceived effort, and self-efficacy (Barhorst et al., 2020; Cracco et al., 2019; Martinez-Calderon et al., 2018; Meier et al., 2016). However, the present cross-sectional findings do not establish that these processes explain the observed between-group differences in CENOAM score.

The absence of floor and ceiling effects indicates that CENOAM scores were distributed across a broad range of the scale in the present sample. However, this finding should not be interpreted as evidence of responsiveness or sensitivity to change over time, which requires longitudinal evaluation.

### 4.5 Practical implications

CENOAM provides a structured assessment of how an individual subjectively appraises a specific observed movement across two related dimensions, Familiarity and Competence–Demand. These scores may provide complementary descriptive information regarding previous familiarity with the observed action and the perceived capability, clarity, difficulty, or effort associated with that action.

However, the present findings do not establish CENOAM as a clinical decision-making instrument. In particular, the results do not demonstrate that CENOAM scores can be used to determine treatment selection, guide graded exposure, personalize intervention content, or monitor clinically meaningful change over time. Likewise, the present cross-sectional associations do not establish that CENOAM scores predict engagement, adherence, treatment response, or functional improvement.

These possibilities should therefore be considered hypotheses for future research. Prospective and longitudinal studies are required before CENOAM scores can be used for individual clinical decision-making or outcome monitoring.

Taken together, the present findings provide initial evidence supporting the psychometric properties of CENOAM for assessing subjective appraisal of a specific observed movement. However, the present validation should be considered an initial step. Further research is required to establish score stability, measurement error, responsiveness, longitudinal interpretability, and the potential clinical utility of CENOAM before the instrument can be recommended for monitoring individual change or guiding treatment decisions.

### 4.6 Limitations

Several limitations should be acknowledged. First, the study employed a cross-sectional design, precluding evaluation of test–retest reliability, measurement error, and responsiveness to change (Prinsen et al., 2018). In addition, the smallest detectable change and the interpretability of longitudinal score changes were not evaluated. Consequently, it is currently unknown whether CENOAM scores are sufficiently stable under unchanged conditions or whether changes in scores over time represent true changes in movement appraisal rather than measurement error. These limitations preclude the use of CENOAM, at this stage, as an established instrument for monitoring individual change or determining clinically meaningful improvement.

Second, although the sample size was adequate for EFA, replication in independent samples is necessary to confirm the stability of the factor structure.

Third, an important limitation of the present study is that measurement invariance between participants with CLBP and asymptomatic participants was not formally established. Consequently, the pooled factor solution should not be interpreted as demonstrating that CENOAM measures its underlying dimensions identically in both groups, and the observed between-group differences cannot be interpreted as equivalent latent mean differences. Although the present sample included 140 participants per group, the study was not prospectively designed or specifically powered as a formal multigroup validation study. This should be considered when interpreting the known-groups findings.

Fourth, although redundancy analyses suggested some overlap between items, further refinement may be necessary to optimize parsimony while preserving construct coverage.

In addition, the CLBP and asymptomatic groups were recruited from different settings and differed in several demographic characteristics. Although the observed CENOAM score differences remained substantial after adjustment for age, sex, BMI, and educational level, residual confounding cannot be excluded. The clinical characterization of the CLBP sample was also limited to the pain-related variables collected in the study, including current pain intensity, chronic pain self-efficacy, pain catastrophizing, and fear of movement. Pain duration, disability, current treatment status, medication use, and work status were not collected. Future validation studies should include these variables to provide a more comprehensive clinical characterization and to examine their potential contribution to CENOAM scores.

Fifth, the present validity evidence was obtained using a single standardized motor stimulus. Therefore, CENOAM scores should not currently be interpreted as stable individual characteristics that necessarily generalize across different observed actions. Rather, they reflect an individual’s appraisal of the specific observed motor stimulus. Although standardizing the stimulus allowed interindividual differences in appraisal to be examined under common observation conditions, the present design does not allow person, stimulus, person-by-stimulus, and residual sources of score variance to be separated. Future studies should administer the CENOAM to the same participants across multiple standardized motor actions to determine the extent to which scores generalize across stimuli and to quantify the relative contribution of these different sources of variance. Additionally, variability in cognitive load during observation tasks—particularly in chronic pain populations—may influence response accuracy, and greater control over stimulus characteristics (e.g., perspective, speed, congruence) may help reduce measurement noise (Bach et al., 2011; Decroix and Kalénine, 2019).

An additional limitation is that the present study assessed subjective movement appraisal and did not include an objective measure of participants’ ability to execute the observed action. Consequently, the extent to which perceived competence or demand corresponds to actual motor performance cannot be determined from the present data.

Finally, incorporation of neurophysiological measures (e.g., EEG or fMRI) could clarify how the identified dimensions relate to underlying neural mechanisms of action simulation and inference (Brass et al., 2007; Urgen et al., 2022).

### 4.7 Future directions

Future research should prioritize replication of the factor structure in independent samples.

A priority for the next stage of CENOAM validation is a prospectively designed multigroup study examining measurement equivalence across clinical and asymptomatic populations. Such research should first establish the adequacy and stability of the measurement model separately within each population before sequentially examining configural, metric, and scalar invariance and, where necessary, partial invariance or item-level sources of non-invariance. Establishing scalar invariance will be particularly important before between-group differences can be interpreted as differences in latent CENOAM constructs.

Longitudinal studies are needed to establish temporal stability and sensitivity to change. Future studies should specifically evaluate test–retest reliability and measurement error under stable conditions and establish the smallest detectable change for CENOAM scores. Longitudinal studies should subsequently examine responsiveness to changes in movement appraisal and determine how score changes should be interpreted over time. Where appropriate external anchors are available, future research should also investigate thresholds for clinically meaningful change. Further refinement of the instrument, including expansion of the item pool, may improve construct coverage and support the development of shorter versions for different contexts.

Future studies should examine CENOAM scores alongside objective measures of motor execution and functional performance. In particular, prospective designs should determine whether movement-specific CENOAM appraisals predict subsequent action execution, engagement with action-observation interventions, treatment response, or functional outcomes. Such studies would help establish the extent to which subjective appraisal and actual motor capacity converge or diverge.

In addition, future studies should incorporate more controlled and ecologically valid stimuli, including standardized video sets or immersive virtual reality environments, enabling systematic manipulation of perspective and task complexity (Castro and Schenke, 2024).

Experimental research is also warranted to determine whether interventions tailored according to individual profiles lead to improved adherence and functional outcomes. In this context, evidence suggests that congruence between the observer’s motor repertoire and the observed action enhances simulation-based encoding processes (Bouazzaoui et al., 2025), supporting the potential value of personalized approaches.

Another important direction is to extend CENOAM validation beyond musculoskeletal pain populations. Given the relevance of action observation to neurorehabilitation, future studies should examine the measurement properties of CENOAM in neurological populations, including individuals experiencing pain associated with acquired brain injury. In these populations, movement appraisal may reflect interactions among motor impairment, previous motor experience, pain-related processes, perceived competence, and the characteristics of the observed action. Dedicated validation studies are therefore required to determine whether the dimensional structure and score interpretation identified in the present study generalize to neurological populations. Such research may ultimately clarify whether movement-appraisal assessment can provide useful complementary information for the design of individualized action-observation–based interventions in neurorehabilitation.

### 4.8 Conclusion

This study reports the development of the CENOAM and provides initial evidence supporting the validity and reliability of its scores. The instrument demonstrated a robust two-dimensional structure, supported by converging evidence from multiple analytical approaches. Scores showed high internal consistency, absence of floor and ceiling effects, theoretically coherent associations with relevant constructs, and strong discriminative capacity between individuals with CLBP and asymptomatic participants.

These findings position the instrument as a promising tool for assessing how individuals appraise observed movements, with potential relevance for both research and clinical practice. Further studies are needed to establish its longitudinal properties, measurement invariance, and responsiveness to change, as well as its role in guiding personalized rehabilitation strategies.

## Data Availability

The raw data supporting the conclusions of this article will be made available by the authors, without undue reservation.
