# Supplementary material for: Motor action observation evaluation questionnaire: development and initial validity evidence for the scores in people with and without chronic low back pain
Source: Front Psychol. 2026 Oct 15;17:1887879. doi: 10.3389/fpsyg.2026.1887879 (PMC13716616; doi:10.3389/fpsyg.2026.1887879)
Supplement: Supplementary file 1 [file Table_1.docx]

Supplementary Material

**Supplementary Table S1**: item-level correlation matrix — asymptomatic participants.

| **Item** | **CENOAM 1** | **CENOAM 2** | **CENOAM 3** | **CENOAM 4** | **CENOAM 5** | **CENOAM 6** | **CENOAM 7** |
| --- | --- | --- | --- | --- | --- | --- | --- |
| **CENOAM 1** | 1.00 | −.34 | −.34 | .50 | .70 | −.24 | .57 |
| **CENOAM 2** | −.34 | 1.00 | .74 | −.39 | −.33 | .71 | −.30 |
| **CENOAM 3** | −.34 | .74 | 1.00 | −.38 | −.40 | .64 | −.25 |
| **CENOAM 4** | .50 | −.39 | −.38 | 1.00 | .63 | −.19 | .43 |
| **CENOAM 5** | .70 | −.33 | −.40 | .63 | 1.00 | −.18 | .53 |
| **CENOAM 6** | −.24 | .71 | .64 | −.19 | −.18 | 1.00 | −.30 |
| **CENOAM 7** | .57 | −.30 | −.25 | .43 | .53 | −.30 | 1.00 |

**Note.** Values are Pearson correlation coefficients. CENOAM = Questionnaire for the Assessment of Motor Action Observation.
