# Supplementary material for: Motor action observation evaluation questionnaire: development and initial validity evidence for the scores in people with and without chronic low back pain
Source: Front Psychol. 2026 Oct 15;17:1887879. doi: 10.3389/fpsyg.2026.1887879 (PMC13716616; doi:10.3389/fpsyg.2026.1887879)
Supplement: Supplementary file 3 [file Table_3.docx]

Supplementary Material

**Supplementary Table S3**: Correlations among CENOAM Total, Familiarity, and Competence–Demand, separately by group.

| **Group** | **Total–Familiarity** | **Total–Competence–Demand** | **Familiarity–Competence–Demand** |
| --- | --- | --- | --- |
| **Asymptomatic participants** | .598 | .488 | .334 |
| **Participants with CLBP** | .609 | .806 | .696 |

Note. Values are Pearson correlation coefficients. CLBP = chronic low back pain; CENOAM = Questionnaire for the Assessment of Motor Action Observation.
