# Supplementary material for: Motor action observation evaluation questionnaire: development and initial validity evidence for the scores in people with and without chronic low back pain
Source: Front Psychol. 2026 Oct 15;17:1887879. doi: 10.3389/fpsyg.2026.1887879 (PMC13716616; doi:10.3389/fpsyg.2026.1887879)
Supplement: Supplementary file 4 [file Table_4.docx]

Supplementary Material

**Supplementary Table S4.** Pattern and Structure Matrices for the Two-Factor Exploratory Factor Analysis of the CENOAM

| **Item** | **Pattern: Familiarity** | **Pattern: Competence–Demand** | **Structure: Familiarity** | **Structure: Competence–Demand** |
| --- | --- | --- | --- | --- |
| CENOAM 1 | −.111 | .785 | .225 | .738 |
| CENOAM 2 | .925 | −.010 | .921 | .386 |
| CENOAM 3 | .921 | −.046 | .901 | .348 |
| CENOAM 4 | −.064 | .682 | .228 | .655 |
| CENOAM 5 | .383 | .642 | .657 | .806 |
| CENOAM 6 | .863 | .063 | .890 | .433 |
| CENOAM 7 | .010 | .751 | .331 | .755 |

Note. Exploratory factor analysis was performed using minimum residual (MINRES) extraction with direct oblimin rotation. Pattern coefficients represent the unique association of each item with each factor after accounting for the correlation between factors, whereas structure coefficients represent the zero-order correlations between items and factors. The correlation between Familiarity and Competence–Demand was r = .428.
