# Supplementary material for: Motor action observation evaluation questionnaire: development and initial validity evidence for the scores in people with and without chronic low back pain
Source: Front Psychol. 2026 Oct 15;17:1887879. doi: 10.3389/fpsyg.2026.1887879 (PMC13716616; doi:10.3389/fpsyg.2026.1887879)
Supplement: Supplementary file 5 [file Table_5.docx]

Supplementary Material

**Supplementary Table S5.** Relations between CENOAM total and subscale scores and external variables in asymptomatic participants and participants with CLBP

A. **Asymptomatic participants (n = 140)**

| **CENOAM score** | **External variable** | **r** | **95% CI** | **p** |
| --- | --- | --- | --- | --- |
| Total | MIQ-R Kinesthetic | .112 | [−.055, .273] | .1885 |
| Total | MIQ-R Visual | .536 | [.407, .645] | < .001 |
| Total | MIQ-R Total | .391 | [.240, .523] | < .001 |
| Total | IPAQ Physical Activity | .485 | [.347, .603] | < .001 |
| Total | CPSS | .166 | [−.000, .323] | .0503 |
| Total | PCS Total | −.282 | [−.428, −.122] | < .001 |
| Total | TSK-11 | −.242 | [−.392, −.079] | .0040 |
| Familiarity | MIQ-R Kinesthetic | .318 | [.160, .459] | < .001 |
| Familiarity | MIQ-R Visual | .562 | [.437, .666] | < .001 |
| Familiarity | MIQ-R Total | .515 | [.382, .628] | < .001 |
| Familiarity | IPAQ Physical Activity | .519 | [.386, .630] | < .001 |
| Familiarity | CPSS | .340 | [.184, .478] | < .001 |
| Familiarity | PCS Total | −.164 | [−.322, .002] | .0523 |
| Familiarity | TSK-11 | −.359 | [−.495, −.205] | < .001 |
| Competence–Demand | MIQ-R Kinesthetic | .205 | [.040, .358] | .0154 |
| Competence–Demand | MIQ-R Visual | .446 | [.303, .570] | < .001 |
| Competence–Demand | MIQ-R Total | .384 | [.233, .517] | < .001 |
| Competence–Demand | IPAQ Physical Activity | .404 | [.255, .534] | < .001 |
| Competence–Demand | CPSS | .359 | [.205, .495] | < .001 |
| Competence–Demand | PCS Total | −.123 | [−.283, .044] | .1484 |
| Competence–Demand | TSK-11 | −.388 | [−.520, −.237] | < .001 |

B. **Participants with CLBP (n = 140)**

| **CENOAM score** | **External variable** | **r** | **95% CI** | **p** |
| --- | --- | --- | --- | --- |
| Total | NPRS | −.167 | [−.324, −.001] | .0483 |
| Total | MIQ-R Kinesthetic | .209 | [.045, .363] | .0130 |
| Total | MIQ-R Visual | .422 | [.276, .550] | < .001 |
| Total | MIQ-R Total | .377 | [.225, .511] | < .001 |
| Total | IPAQ Physical Activity | .098 | [−.069, .259] | .2508 |
| Total | CPSS | .626 | [.513, .717] | < .001 |
| Total | PCS Total | −.466 | [−.586, −.325] | < .001 |
| Total | TSK-11 | −.517 | [−.629, −.384] | < .001 |
| Familiarity | NPRS | −.316 | [−.458, −.158] | < .001 |
| Familiarity | MIQ-R Kinesthetic | .444 | [.300, .568] | < .001 |
| Familiarity | MIQ-R Visual | .468 | [.328, .589] | < .001 |
| Familiarity | MIQ-R Total | .507 | [.372, .620] | < .001 |
| Familiarity | IPAQ Physical Activity | .382 | [.231, .515] | < .001 |
| Familiarity | CPSS | .578 | [.456, .679] | < .001 |
| Familiarity | PCS Total | −.506 | [−.620, −.371] | < .001 |
| Familiarity | TSK-11 | −.647 | [−.734, −.539] | < .001 |
| Competence–Demand | NPRS | −.220 | [−.372, −.056] | .0091 |
| Competence–Demand | MIQ-R Kinesthetic | .279 | [.118, .425] | < .001 |
| Competence–Demand | MIQ-R Visual | .468 | [.328, .588] | < .001 |
| Competence–Demand | MIQ-R Total | .437 | [.293, .562] | < .001 |
| Competence–Demand | IPAQ Physical Activity | .123 | [−.044, .283] | .1476 |
| Competence–Demand | CPSS | .622 | [.509, .715] | < .001 |
| Competence–Demand | PCS Total | −.567 | [−.670, −.443] | < .001 |
| Competence–Demand | TSK-11 | −.522 | [−.633, −.390] | < .001 |

**Note.** Values are Pearson correlation coefficients with 95% confidence intervals. Analyses were conducted separately in asymptomatic participants and participants with CLBP. Pain intensity (NPRS) was evaluated only in the CLBP group. Physical activity corresponds to the continuous IPAQ-S score expressed as MET-min/week. No adjustment for multiple comparisons was applied. CENOAM = Questionnaire for the Assessment of Motor Action Observation; MIQ-R = Movement Imagery Questionnaire–Revised; IPAQ-S = International Physical Activity Questionnaire–Short Form; CPSS = Chronic Pain Self-Efficacy Scale; PCS = Pain Catastrophizing Scale; TSK-11 = Tampa Scale for Kinesiophobia; NPRS = Numeric Pain Rating Scale.
